# Inactivation of Sonic Hedgehog Signaling and Polydactyly in Limbs of Hereditary Multiple Malformation, a Novel Type of *Talpid* Mutant

**DOI:** 10.3389/fcell.2016.00149

**Published:** 2016-12-27

**Authors:** Yoshiyuki Matsubara, Mikiharu Nakano, Kazuki Kawamura, Masaoki Tsudzuki, Jun-Ichi Funahashi, Kiyokazu Agata, Yoichi Matsuda, Atsushi Kuroiwa, Takayuki Suzuki

**Affiliations:** ^1^Division of Biological Science, Graduate School of Science, Nagoya UniversityNagoya, Japan; ^2^Avian Bioscience Research Center, Graduate School of Bioagricultural Sciences, Nagoya UniversityNagoya, Japan; ^3^Laboratory of Animal Breeding and Genetics, Graduate School of Biosphere Science, Hiroshima UniversityHiroshima, Japan; ^4^Institute of Development, Aging and Cancer, Tohoku UniversitySendai, Japan; ^5^Department of Biophysics, Graduate School of Science, Kyoto UniversityKyoto, Japan; ^6^Laboratory of Animal Genetics, Department of Applied Molecular Biosciences, Graduate School of Bioagricultural Sciences, Nagoya UniversityNagoya, Japan

**Keywords:** sonic hedgehog, polydactyly, quail, Hereditary Multiple Malformation

## Abstract

Hereditary Multiple Malformation (HMM) is a naturally occurring, autosomal recessive, homozygous lethal mutation found in Japanese quail. Homozygote embryos (*hmm*^−/−^) show polydactyly similar to *talpid*^2^ and *talpid*^3^ mutants. Here we characterize the molecular profile of the *hmm*^−/−^ limb bud and identify the cellular mechanisms that cause its polydactyly. The *hmm*^−/−^ limb bud shows a severe lack of sonic hedgehog (SHH) signaling, and the autopod has 4 to 11 unidentifiable digits with syn-, poly-, and brachydactyly. The Zone of Polarizing Activity (ZPA) of the *hmm*^−/−^ limb bud does not show polarizing activity regardless of the presence of SHH protein, indicating that either the secretion pathway of SHH is defective or the SHH protein is dysfunctional. Furthermore, mesenchymal cells in the *hmm*^−/−^ limb bud do not respond to ZPA transplanted from the normal limb bud, suggesting that signal transduction downstream of SHH is also defective. Since primary cilia are present in the *hmm*^−/−^ limb bud, the causal gene must be different from *talpid*^2^ and *talpid*^3^. In the *hmm*^−/−^ limb bud, a high amount of GLI3A protein is expressed and GLI3 protein is localized to the nucleus. Our results suggest that the regulatory mechanism of GLI3 is disorganized in the *hmm*^−/−^ limb bud.

## Introduction

Avian mutants have often been used to study developmental mechanisms, especially embryonic pattern formation. Some of the most well studied mutant strains in chickens are the *talpids* (*talpid, talpid*^2^, and *talpid*^3^) (Cole, 1942). These three *talpid* mutants are naturally occurring and were independently discovered. The original *talpid* mutation has since been lost, but *talpid*^2^ and *talpid*^3^ are still maintained in the UK and the USA. Intriguingly, these mutants share a unique phenotype characterized by polydactyly, craniofacial abnormality, autosomal recessive inheritance, and embryonic lethality. The gene responsible for *talpid*^2^ was identified as *C2CD3* (Chang et al., 2014), whereas the gene responsible for *talpid*^3^ is *KIAA0586* (Davey et al., 2006). These causal genes are both involved in the formation of primary cilia (Yin et al., 2009; Chang et al., 2014).

The primary cilium is thought to be necessary for intermediate sonic hedgehog (SHH) signaling because it provides a location for the processing of the transcriptional factor GLI3 (Besse et al., 2011). SHH is secreted from the Zone of Polarizing Activity (ZPA), which is located at the posterior edge of the limb bud, and determines the limb's anterior-posterior (AP) axis (Riddle et al., 1993). In the absence of SHH, GLI3 is located in the primary cilium and is phosphorylated by protein kinase A (Wang et al., 2000; Hsu et al., 2011). Phosphorylated GLI3 is ubiquitinated, resulting in partial degradation (Bhatia et al., 2006). This short form of GLI3, called GLI3R, inhibits the transcription of target genes (Wang et al., 2000). In the presence of SHH, GLI3 is maintained in a long activator form called GLI3A (Litingtung et al., 2002). GLI3A induces expression of target genes such as *Patched1* (*Ptch1*).

Interestingly, although both C2CD3 and KIAA0586 proteins are necessary for the ciliogenesis pathway to proceed, the *talpid*^2^ and *talpid*^3^ mutants indicate they have different impacts on SHH signaling. In the *talpid*^2^ limb bud, SHH signaling is constitutively activated by the upregulation of GLI3A, which causes anterior expansion of *Ptch1, Bmp4, Fgf4*, and *Hoxd13* expression (Rodriguez et al., 1996; Caruccio et al., 1999). In contrast, SHH signaling is abolished in the *talpid*^3^ limb bud leading to downregulation of *Ptch1* and *Gli1* expression, but GLI3A is still upregulated (Davey et al., 2006) as in the *talpid*^2^ mutant. It is known that in *Shh* deficient conditions only GLI3R is present, resulting in the formation of only digit 1 in the hindlimb and undetectable expression of *Ptch1* and *Gli1* (Chiang et al., 2001). The *talpid*^3^ mutant is thought to be similar, but it is still unclear why the SHH signaling pathway is defective in the *talpid*^3^ mutant despite up-regulation of GLI3A.

The HMM mutant was reported as a similar mutant phenotype to *talpid* in 1998 (Tsudzuki et al., 1998). It is a naturally occurring, autosomal recessive, homozygous lethal Japanese quail mutant. The gene responsible for *hmm* is still unknown. Homozygote embryos show polydactyly and shortened lower and upper beaks, which is slightly different from the *talpid*^2^ mutant phenotype of an extended lower beak compared to the upper beak (Chang et al., 2014). The HMM mutant also does not display the subcutaneous edema and hemorrhage over the thigh and neck regions found in the *talpid*^2^ and *talpid*^3^ mutants (Tsudzuki et al., 1998). Based on these observations, the developmental causes of the HMM mutant are likely different from the *talpid*^2^ and *talpid*^3^ mutants.

Here we characterize the molecular profile of the HMM mutant and perform a functional analysis of the cellular mechanisms that cause the mutant phenotype. Gene expression patterns indicate that SHH signaling is defective in the homozygous HMM mutant (*hmm*^−/−^), similar to the *talpid*^3^ mutant. However, the limb bud in the *hmm*^−/−^ embryo still has anterior-posterior polarity with restricted anterior marker gene expression. This is different from the limb bud patterning in *talpid*^2^ and *talpid*^3^. Furthermore, we found that the ZPA in the *hmm*^−/−^ limb bud does not show polarizing activity regardless of the presence of SHH protein expression. The primary cilium was present however, and we observed a high amount of GLI3A protein in the *hmm*^−/−^ limb bud. These results indicate that different molecular pathways than *talpid*^2^ and *talpid*^3^ are defective in the *hmm*^−/−^ limb bud.

## Materials and methods

### Embryos

The fertilized HMM mutant quail eggs were provided by Avian Bioscience Research center (ABRC) at Nagoya University. Embryos were staged according to Ainsworth et al. (2010). The HMM mutant shows autosomal recessive inheritance, although the causal gene, *hmm*, is still unknown. Heterozygous embryos showed no phenotype and were indistinguishable from wild-type embryos. Therefore we used a mixture of wild-type and heterozygous embryos as a control. Experimental procedures for isolating embryos were performed in accordance with guidelines set forth by the Regulations on Animal Experiments at Nagoya University. The embryo research was approved by Nagoya University Animal Experiment Committee (approval number 17).

### Visualization of 3D image of the limb bud by OPT scanner

Limb buds were fixed with 4% PFA overnight and then embedded in 1% low-melting agarose (Lonza). Excess agarose around the limb was removed with a razor blade. After that, agarose containing the limb buds was attached to the swivel base with Loctite for optical projection tomography (OPT) scanning (Henkel). Limb buds were treated with 100% MeOH for 3 h, and samples cleared in a 1:2 solution of benzyl alcohol (Wako): benzyl benzoate (Wako) overnight. A 3D image was taken with the OPT scanner 3001 (Bioptonics) and visualized by Avizo software (Maxnet).

### Skeletal staining and *In situ* hybridization

Victoria blue staining was performed as described previously (Suzuki et al., 2008). Embryos were dissected in PBS and fixed in 10% Formalin overnight at room temperature. Embryos were stained overnight with 1% Victoria blue (Sigma) solution containing 1% HCl, and 70% EtOH. Embryos were washed overnight with 1% HCl in 70% EtOH solution following overnight treatment with 100% methylsalicylate to render them transparent.

*In situ* hybridization was performed as described previously (Suzuki et al., 2008). The following probes were used for *in situ* hybridizations: *Hoxa13* (Yokouchi et al., 1991), *Hoxd13* (Nelson et al., 1996), *Fgf8, Gli3, Alx4, Lhx9, Ptch2*, and *Bmp2* (kindly gifted by Dr. John. F. Fallon, University of Wisconsin-Madison), *Hand2* (kindly gifted by Dr. Kazuko Koshiba-Takeuchi, University of Tokyo), *Shh, Gli1*, and *Ptch1* (kindly gifted by Dr. Yuki Sato, Kyushu university), *Pax6* (kindly gifted by Dr. Yoshio Wakamatsu, Tohoku university), *Pax3, Pax7*, and *Dbx2* (kindly gifted by Dr. Harukazu Nakamura, Tohoku university), *Islet1* (589–1551 bp, GenBank NM_205414), and *MyoD* (155–1051 bp, GenBank NM_204214).

### Implantation of the ZPA

The ZPA was isolated from the posterior side of the donor limb bud with a sharpened tungsten needle. Isolated ZPA was placed in ice-cold Tyrode solution (137 mM NaCl, 2.7 mM KCl, 1 mM MgCl_2_, 1.8 mM CaCl_2_, 0.2 mM Na_2_HPO_4_, 12 mM NaHCO_3_, 5.5 mM d-glucose) and divided into several pieces. A piece of the ZPA was stained by squirting it with DiI solution (1% DiI dissolved with 70% EtOH) in Tyrode solution, and it was implanted at the anterior side of the host limb bud with a tungsten needle.

### Immunohistochemistry

Primary embryonic fibroblasts were isolated from the back region between the forelimb and the hindlimb at St. 35. Back tissues were dissected in PBS. After tissues were minced with a razor blade, they were incubated in 0.25% trypsin-EDTA solution (Wako) for 15 min at 37°C. An equal volume of 100% fetal calf serum was added. The supernatant of the cell suspension was plated on 3.5-cm glass-base tissue culture dish (IWAKI). The next day, cells were fixed with 4% PFA for 10 min at room temperature.

Limb buds were dissected in ice-cold PBS and fixed with 4% PFA for 15 min on ice. The PFA solution was immediately removed and fresh ice-cold PBS was added. The limb buds were treated with 30% sucrose in PBS overnight at 4°C and then embedded in compound for frozen sections (Leica). Samples were then sectioned by cryostat for immunohistochemistry.

Cells in frozen sections or fixed primary fibroblasts were permeabilized by treating with 0.2% TritonX-100 (Wako) for 20 min at room temperature, and then blocked with 3% BSA in PBS for 30 min. Anti-SHH antibody 5E1 (1:100) (DSHB), Anti-GLI3 antibody (1:100) (Santa Cruz sc-20688), and anti-acetylated Tubulin clone 6-11B-1 (1:1000) (Sigma) were diluted in 3% BSA/PBS solution. Samples were incubated overnight at 4°C with the primary antibody. The next day, samples were incubated with the secondary antibody (Alexa Fluor 488) (Thermo Fisher Scientific) diluted at 1:500 in 3% BSA/PBS solution for 2 h at room temperature. After 1 μg/ml of DAPI in PBS was added to the samples for 15 min, the samples were mounted with fluorescein mounting medium (Dako) and fluorescent images were taken with an Olympus FV1000 confocal microscope.

### Western blot analysis

Fertilized eggs were incubated for 3.5 days. The limb buds of St. 23 embryos were isolated in ice-cold PBS with a tungsten needle and then collected in 1.5 mL collection tubes. After solubilizing the cells with 200 μl of lysis buffer (50 mM HEPES (pH 7.4), 150 mM NaCl, 10 mM EDTA, 1% Triton X-100, 200 mM sodium fluoride, 10% glycerol, 20 mM sodium pyrophosphate, 2 mM phenylmethane sulfonyl fluoride, 4 mM Na_3_VO_4_, 0.1 mg/ml leupeptin, and 15 mM benzamidine), the cell lysate was centrifuged at 14,000 g for 10 min. The supernatant was assayed for protein content using a Bio-Rad protein assay kit. The proteins were then resolved with SDS-polyacrylamide gel electrophoresis and electrotransferred to polyvinylidene difluoride membranes. After blocking with 5% (w/v) non-fat dry milk in Tris-buffered saline-Tween buffer (20 mM Tris (pH 7.6), 0.14 M NaCl, and 0.1% (w/v) Tween 20), the membranes were treated with primary antibodies, Anti-GLI3 antibody (1:1000) (Santa Cruz sc-20688), and Anti-αTubulin clone B-5-1-2 (1:2500) (Sigma). The proteins were visualized using an ECL Western blotting detection system.

## Results

### Expanded expression of *Fgf8* at the AER is observed in the *hmm^−/−^* limb bud

**Figure 1:**
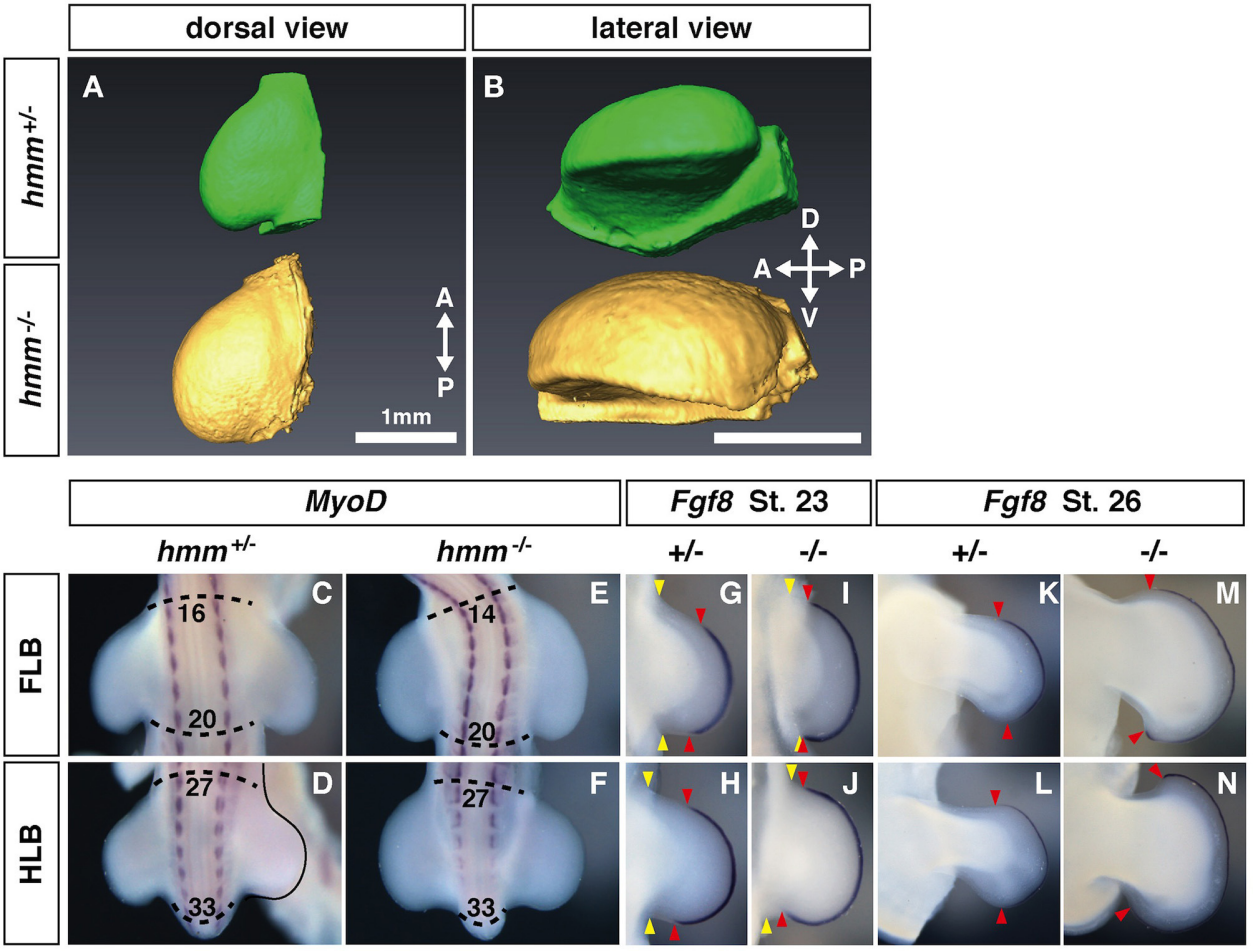
**Expression of ***Fgf8*** at the apical ectodermal ridge (AER) is expanded along the anterior-posterior (AP) axis in the ***hmm***^**−/−**^ limb bud. (A,B)** Morphology of the limb at St. 22 as scanned by OPT. Dorsal view **(A)** and lateral view **(B)** of the *hmm*^+/−^ (green) and *hmm*^−/−^ (yellow) hindlimb buds. The scale bar indicates 1 mm. **(C–F)** *In situ* hybridization of *MyoD*. The dotted line shows the anterior and posterior end of the limb buds. Numbers indicate the position of the somite starting with the first somite. **(G–N)** *In situ* hybridization of *Fgf8*. Yellow arrowheads indicates the boundary between the limb bud and the body wall. Red arrowheads indicate the anterior and posterior boundaries of *Fgf8* expression at the AER. A, anterior; P, posterior; D, dorsal; V, ventral; FLB, forelimb bud; HLB, hindlimb bud.

We previously reported that the *hmm*^−/−^ embryo shows syndactylous polydactyly (Tsudzuki et al., 1998). It is still unclear how these phenotypes are induced in *hmm*^−/−^ limb bud. In both *talpid*^2^ and *talpid*^3^ mutants, the limb bud is wider in size along the AP axis before cartilage condensation starts (Francis-West et al., 1995; Caruccio et al., 1999). This wider limb bud leads to expansion of the autopod, resulting in polydactyly (Litingtung et al., 2002). Therefore, we first examined the shape of the limb bud in the *hmm*^−/−^ embryo. To compare the shape of the entire limb bud between the mutant and wild-type, we took OPT images (Sharpe et al., 2002) of the hind limb at St. 23, after the limb bud emerged from the body wall (Figures 1A,B). The *hmm*^−/−^ limb bud was slightly wider than the *hmm*^+/+;+/−^ (simplified hereafter as *hmm*^+/−^) limb bud along the AP axis (Figure 1A). The *hmm*^−/−^ limb bud was also expanded along the dorso-ventral (DV) axis compared to the *hmm*^+/−^ limb bud (Figure 1B). To elucidate the mechanisms underlying the expansion of the limb bud along the AP axis, we compared the limb field's AP width between *hmm*^+/−^ and *hmm*^−/−^ embryos. We found that the anterior boundary of the *hmm*^−/−^ forelimb bud (FLB) was expanded cranially compared to that of the *hmm*^+/−^ FLB (Figures 1C,E). The AP width of the hindlimb bud (HLB) appeared to be the same in both *hmm*^+/−^ and *hmm*^−/−^ embryos (Figures 1D,F), implying that a different mechanism is involved in the expansion of the hindlimb along the AP axis. In the *talpid*^2^ mutant, the expansion of *Fgf8* expression at the apical ectodermal ridge (AER) along the AP axis is observed along with formation of a wider limb bud (Caruccio et al., 1999). Therefore, we checked the expression of *Fgf8* at St. 23 and St. 26 in the *hmm*^−/−^ limb bud, and found that it was expanded into both the anterior and posterior ends close to the body wall (Figures 1G–J). Expansion of *Fgf8* expression was continued into St. 26, along with wider autopod formation in the *hmm*^−/−^ embryo (Figures 1K–N). Taken together, these results suggest that mesenchymal cells of the *hmm*^−/−^ limb bud propagate more than in the wild-type and give rise to a wider limb bud with an extended AER along the AP axis. In addition, the anterior boundary of the forelimb field is expanded in the *hmm*^−/−^ embryo when the FLB is initiated.

### Anterior-posterior polarity is disrupted in the *hmm^−/−^* limb bud

**Figure 2:**
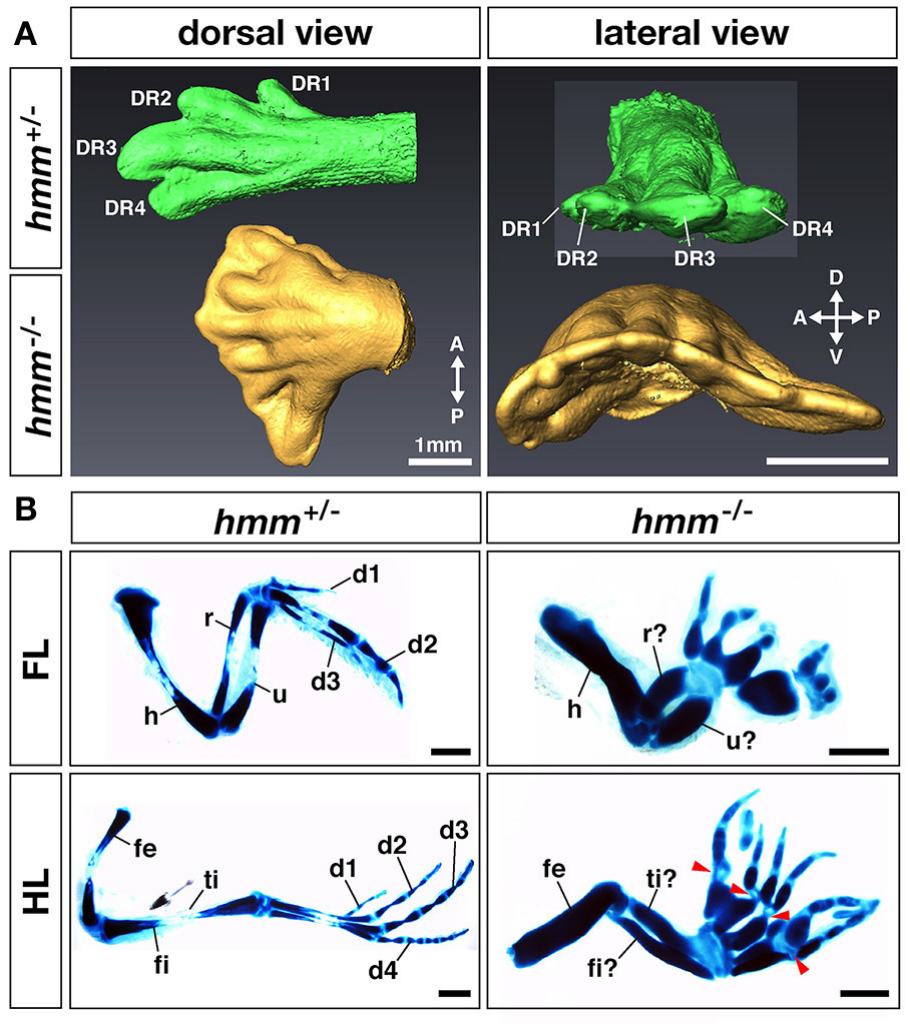
**Anterior-posterior polarity is disrupted in the ***hmm***^**−/−**^ limb bud. (A)** Morphology of the limb at St. 34 as scanned by OPT. Dorsal view and lateral view of the *hmm*^+/−^ (green) and *hmm*^−/−^ (yellow) hindlimb buds are shown. The scale bar indicates 1 mm. **(B)** The skeletal pattern of the limb at St. 36. All images are oriented with the anterior side up and the posterior side down. Red arrowheads indicate immature metatarsal-phalangeal joints. The scale bar indicates 1 mm. A, anterior; P, posterior; D, dorsal; V, ventral; DR, digital ray; FL, forelimb; HL, hindlimb; d1-d4, digit 1-digit 4; h, humerus; r, radius; u, ulna; fe, femur; ti, tibia; fi, fibula.

In the autopod of the *hmm*^−/−^ embryo many of the digits are shortened during development (Tsudzuki et al., 1998). We therefore analyzed detailed patterns of polydactylous digits along the AP axis. In the *hmm*^+/−^ HLB, digital rays 1, 2, 3, and 4 are formed correctly to their unique full lengths along the AP axis (Figure 2A). In contrast, we observed indistinguishable shortened digital rays in the *hmm*^−/−^ HLB. We also found webbing along the AP axis of the digital ray sequence in the *hmm*^−/−^ HLB. These results imply that anterior-posterior polarity is disrupted in the *hmm*^−/−^ limb bud.

In order to identify the specific region where anterior-posterior polarity is disrupted, we performed victoria blue staining to visualize condensing cartilage at St. 35 (Figure 2B). In the forelimb, the *hmm*^−/−^ embryo has four to eight digits with syn-, poly-, and brachdactyly, whereas the *hmm*^+/−^ embryo has three digits (Table 1). The forelimb of *hmm*^−/−^ has one shortened, thick humerus in the stylopod and an unidentifiable ulna/radius in the zeugopod. The hindlimb of the *hmm*^−/−^ embryo also has one shortened, thick femur in the stylopod and an unidentifiable fibula/tibia in the zeugopod. The autopod of the *hmm*^−/−^ hindlimb has seven to eleven digits (Table 1). Based on the morphological criteria of digit identity (number, size, and shape of the phalanges Suzuki, 2013), we assumed that the *hmm*^−/−^ autopod has lost digit identity. We also found that the metacarpal/metatarsal bones were fused and the metacarpal/metatarsal-phalangeal joints were missing in the *hmm*^−/−^ autopod (Figure 2B arrowhead). We observed the phalangeal joint in both the forelimb and the hindlimb, but its formation was incomplete. These results indicate that anterior-posterior polarity of both the forelimb and the hindlimb is disrupted in the *hmm*^−/−^ embryo.

**Table 1:** **Skeletal pattern of the autopod in HMM mutant**.

**Limb**	**Embryo**	**Number of metacarpals/metatarsals**	**Number of digits**	**Number of phalanges**
Right forelimb	Wild-type	3	3	221
	a	3	8	22211311
	b	3	4	2222
	c	3	7	11121nn
	d	4	5	n2222
	e	4	6	2212nn
	f	2	5	222nn
	g	3	6	22n2nn
Left forelimb	Wlid-type	3	3	221
	a	3	5	22211
	b	3	4	1122
	c	3	5	2122n
	d	4	6	1nn221
	e	2	6	1212nn
	f	4	5	1222n
	g	4	7	1nnn1nn
Right hindlimb	Wild-type	4	4	2345
	a	7	9	333322211
	b	4	8	32213233
	c	5	9	323221321
	d	6	11	32323213423
	e	5	7	3223423
	f	6	8	32233223
	g	5	8	32333424
Left hindlimb	Wild-type	4	4	2345
	a	8	10	3321212332
	b	4	8	32333233
	c	5	8	32333222
	d	6	10	3232133333
	e	6	8	32333333
	f	5	7	3333434
	g	5	8	31424224

*n, non-countable*.

**Figure 3:**
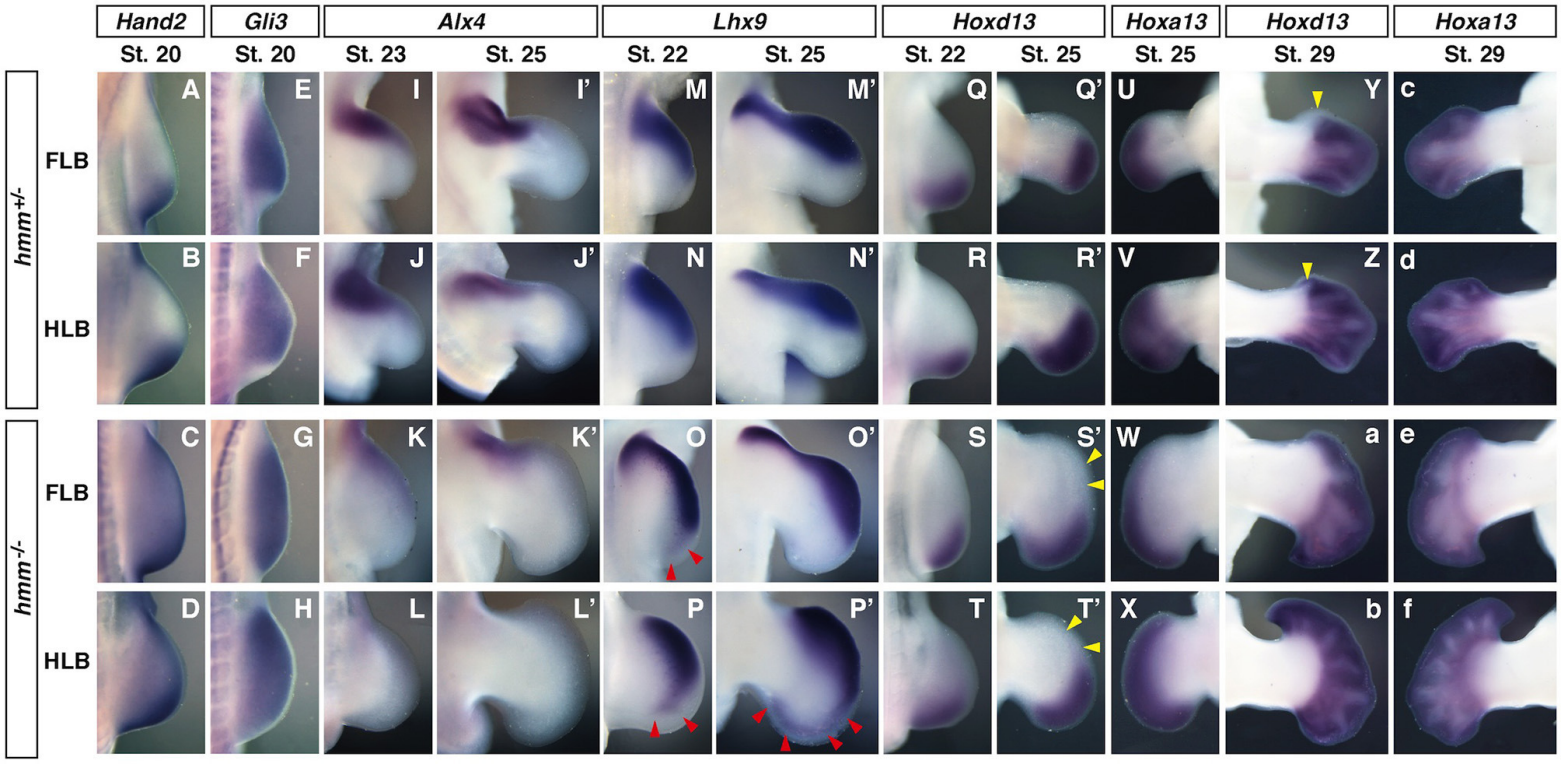
**Anterior-posterior polarity is partially maintained at the limb bud stage in the ***hmm***^**−/−**^ embryo**. *In situ* hybridization of *Hand2* **(A–D)**, *Gli3* **(E–H)**, *Alx4* **(I–L**′**)**, *Lhx9* **(M–P**′**)**, *Hoxd13* **(Q–T**′**,Y–b)** and *Hoxa13* **(U–X,c–f)**. All images are oriented with the anterior side up and the posterior side down. **(O,P,P**′**)** Red arrowheads indicate the expanded expression domain of *Lhx9* on the posterior side. **(S**′**,T**′**)** Yellow arrowheads indicate the region where *Hoxd13* is not expressed in the autopod. **(Y,Z)** Yellow arrowheads indicate the region where *Hoxd13* is not expressed on the anterior side of the digit 1 primordium. FLB, forelimb bud; HLB, hindlimb bud.

To understand the molecular mechanisms of anterior-posterior pattering deficiency in *hmm*^−/−^ limbs, we next examined gene expression patterns of marker genes specifically expressed at the anterior/posterior sides. Genetic antagonism between *Hand2* and *Gli3* is necessary to establish AP polarity at the early limb bud stage (te Welscher et al., 2002a). We found that expression of *Hand2* was restricted at the posterior side in the *hmm*^+/−^ limb bud. In contrast, in the *hmm*^−/−^ limb bud we observed strong expression of *Hand2* at the posterior side and weak expression at the anterior side (Figures 3A–D). In the *hmm*^+/−^ limb bud, *Gli3* was not expressed at the posterior side where *Hand2* was expressed (Figures 3E,F). In contrast, *Gli3* was expressed throughout the limb bud at the early stages in the *hmm*^−/−^ limb bud (Figures 3G,H). *Alx4* was expressed at the anterior mesoderm at St. 23 in the *hmm*^+/−^ limb bud (Figures 3I,J), but its expression was downregulated though still detectable in the *hmm*^−/−^ limb bud (Figures 3K,L). At St. 25, *Alx4* was continuously expressed at the anterior side of the stylopod and zeugopod in the *hmm*^+/−^ limb bud (Figures 3I′,J′). In the *hmm*^−/−^ limb bud, its expression was still observed in the FLB (Figure 3K′) but not detectable in the HLB (Figure 3L′). *Lhx9* was expressed at the anterior side at St. 22 (Figures 3M,N) and at the anterior autopod at St. 25 (Figures 3M′,N′). In contrast to the expression of *Alx4*, expression of *Lhx9* was expanded to the posterior side in the *hmm*^−/−^ limb bud (Figures 3O–P′). In particular, *Lhx9* expression was observed at the posterior end of the *hmm*^−/−^ hindlimb autopod (Figure 3P′). Expression of *Hoxd13* was restricted to the posterior side in the *hmm*^+/−^ limb bud at St. 22 (Figures 3Q,R), and at St. 25 its expression was observed at the posterior mesoderm in the autopod (Figures 3Q′,R′). Expression of *Hoxd13* was similarly restricted to the posterior side in the *hmm*^−/−^ limb bud (Figures 3S–T′). We checked the autopod area for *Hoxa13* expression in the contra-lateral side (Figures 3U–X), and we saw that the autopod expanded along the AP axis in the *hmm*^−/−^ limb bud at St. 25 (Figures 3W,X). However, expression of *Hoxd13* was still restricted to the posterior side (Figures 3S′,T′), the same as in the *hmm*^+/−^ autopod (Figures 3Q′,R′). At St. 29 *Hoxd13* was expressed throughout the *hmm*^+/−^ autopod except on the anterior side of digit 1 (Figures 3Y,Z). However, it was expressed all the way to the anterior end of the *hmm*^−/−^ autopod (Figures 3a,b) along with *Hoxa13* (Figures 3c–f). From these results, we conclude that the *hmm*^−/−^ limb bud partially maintains AP polarity at the limb bud stage (St. 20–25), but loses AP polarity at the late autopod stage (St. 29).

### SHH signaling is reduced in the *hmm^−/−^* limb bud

**Figure 4:**
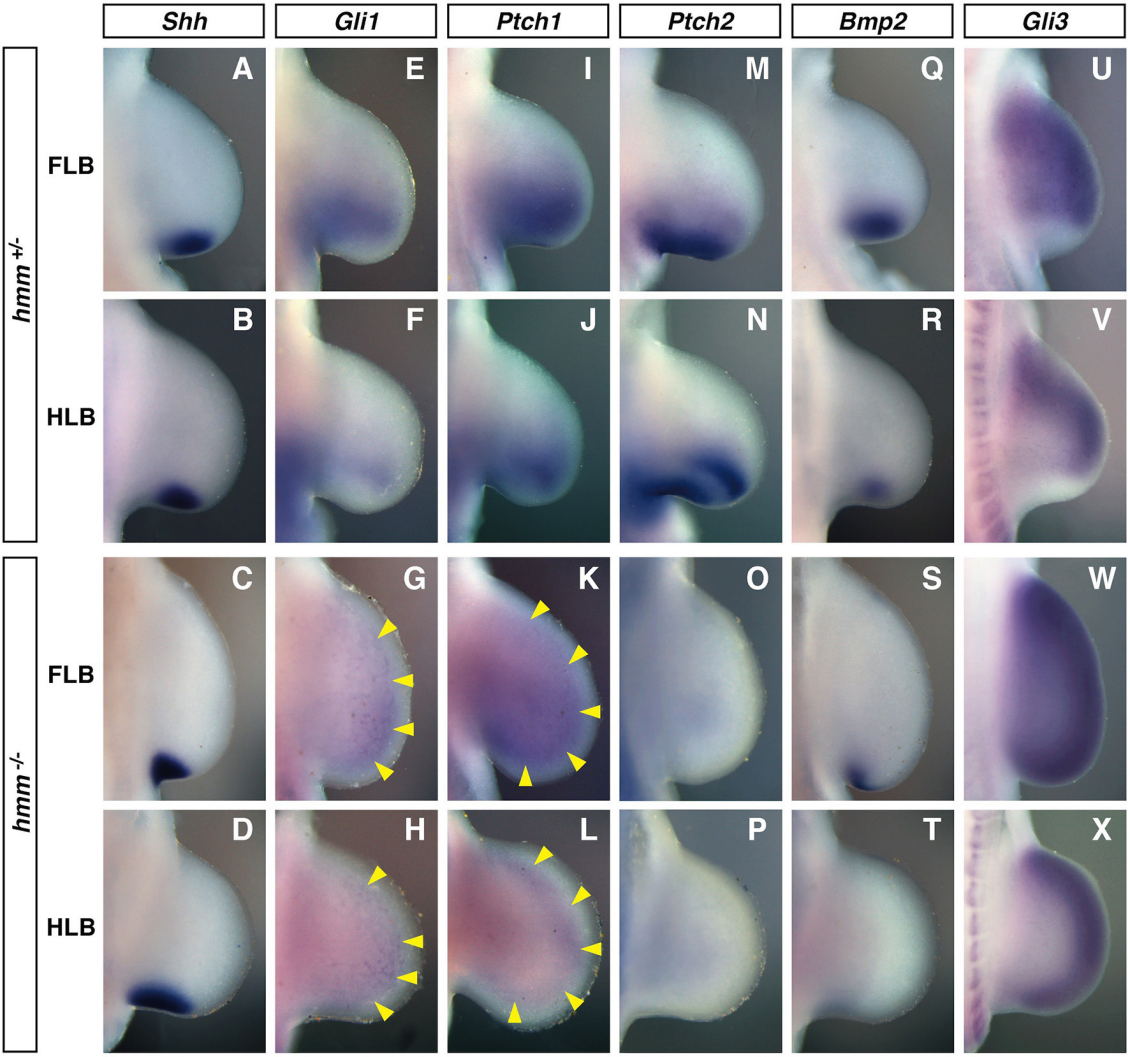
**SHH signaling is reduced in the ***hmm***^**−/−**^ limb bud**. *In situ* hybridization of *Shh* **(A–D)**, *Gli1* **(E–H)**, *Ptch1* **(I–L)**, *Ptch2* **(M–P)**, *Bmp2* **(Q–T)**, and *Gli3* **(U–X)** at St. 22/23. All images are oriented with the anterior side up and the posterior side down. **(G–L)** Yellow arrowheads indicate the weak expression of *Gli1* and *Ptch1*. FLB, forelimb bud; HLB, hindlimb bud.

As described above, AP polarity in the limb bud is disrupted in the *hmm*^−/−^ embryo. It is known that expression of *hand2, Gli3*, and *Alx4* is altered by SHH signaling, which establishes anterior-posterior polarity in the limb (Takahashi et al., 1998; te Welscher et al., 2002a). We therefore examined the expression patterns of target genes downstream of SHH in the limb bud. Expression of *Shh* was restricted to the posterior edge of the *hmm*^+/−^ limb bud (Figures 4A,B). In the *hmm*^−/−^ limb bud, *Shh* was expressed at the posterior mesoderm; however, expression was restricted to a more proximal region of the FLB than in the *hmm*^+/−^ limb bud (Figure 4C). On the other hand, the expression domain of *Shh* was expanded proximally in the HLB (Figure 4D).

*Gli1, Ptch1, Ptch2*, and *Bmp2* are known to be downstream target genes of SHH signaling that are expressed in response to SHH signaling (Chiang et al., 2001). In the *hmm*^+/−^ limb bud, the expression domain of *Gli1* was expanded to the middle part of the limb bud along the anterior-posterior axis (Figures 4E,F). In contrast, *Gli1* was expressed uniformly at very low levels throughout the *hmm*^−/−^ limb bud (Figures 4G,H). Similarly, *Ptch1* was expressed at the posterior half of the *hmm*^+/−^ limb bud (Figures 4I, J), but in the *hmm*^−/−^ limb bud was expressed uniformly at very low levels along the anterior-posterior axis (Figures 4K,L). In the *hmm*^+/−^ limb bud *Ptch2* was expressed at the posterior mesoderm similarly to *Ptch1* (Figures 4M,N), but expression was not detected in the *hmm*^−/−^ limb bud (Figures 4O,P). In the *hmm*^−/−^ limb bud, expression of *Bmp2* was observed at the posterior edge in the FLB only, but in *hmm*^+/−^ limb buds expression was observed at the posterior mesoderm in both the FLB and the HLB (Figures 4Q–T). The expression of *Gli1, Ptch1, Ptch2*, and *Bmp2* is reduced or lost in the limb bud of the *Shh*^−/−^ mouse embryo (Litingtung et al., 2002), therefore SHH signaling must be substantially reduced despite the detection of *Shh* expression in the *hmm*^−/−^ limb bud. Reduction of SHH signaling was also observed in the neural tube of the *hmm*^−/−^ embryo (Figure S1A).

We next examined limb bud expression of *Gli3*, a transcriptional factor that mediates SHH signaling. In the *hmm*^+/−^ limb bud *Gli3* was expressed in the mesenchyme in a complementary pattern to *Shh* expression (Figures 4A,B,U,V). In contrast, in the *hmm*^−/−^ limb bud *Gli3* was strongly expressed throughout the limb bud, including in the *Shh*-expressing region (Figures 4C,D,W,X). We therefore concluded that the syndactylous polydactyly phenotype in the *hmm*^−/−^ limb bud does not result simply from a loss of SHH signaling due to a defect in *Gli3* expression.

### ZPA derived from *hmm^−/−^* limb bud does not have polarizing activity

**Figure 5:**
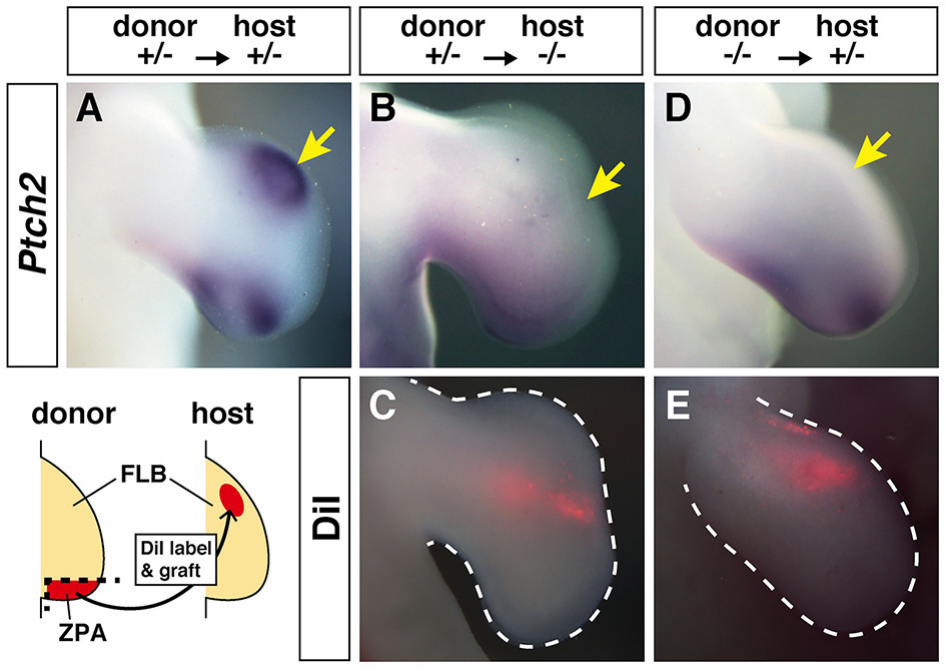
**Zone of Polarizing Activity (ZPA) derived from the ***hmm***^**−/−**^ limb bud does not have polarizing activity. (A,B,D)** *In situ* hybridization of *Ptch2* after ZPA was implanted into the anterior limb bud. **(C,E)** Fluorescence of the implanted ZPA labeled with DiI is shown. The schematic drawing shows the grafting protocol of ZPA from the donor limb bud (*hmm*^+/−^ or *hmm*^−/−^) to the host limb bud (*hmm*^+/−^ or *hmm*^−/−^). The yellow arrows indicate the position of the implanted ZPA. FLB, forelimb bud.

In *hmm*^−/−^ embryos, cells in the limb bud showed a reduction in SHH signaling despite expression of *Shh* (Figure 4). We therefore hypothesized that cells in the *hmm*^−/−^ embryo might have lost SHH protein activity or the ability to respond to SHH protein. To test these hypotheses, we analyzed the polarizing activity of the *hmm*^−/−^ limb bud. When we grafted ZPA from the *hmm*^+/−^ FLB to the anterior side of the *hmm*^+/−^ FLB at St. 20, strong ectopic *Ptch2* expression was observed (83%, *n* = 6) (Figure 5A). In contrast, implantation of ZPA from the *hmm*^+/−^ FLB to the *hmm*^−/−^ FLB did not induce ectopic *Ptch2* expression (0%, *n* = 4) (Figures 5B,C). We next grafted ZPA from the *hmm*^−/−^ FLB to the *hmm*^+/−^ FLB. Unexpectedly, ectopic *Ptch2* expression was not induced (0%, *n* = 8) (Figures 5D,E). These results show that mesenchymal cells in the *hmm*^−/−^ limb bud do not respond to SHH protein. Furthermore, despite the presence of *Shh* expression (Figures 4C,D), ZPA derived from the *hmm*^−/−^ limb bud cannot induce expression of SHH signaling downstream targets.

**Figure 6:**
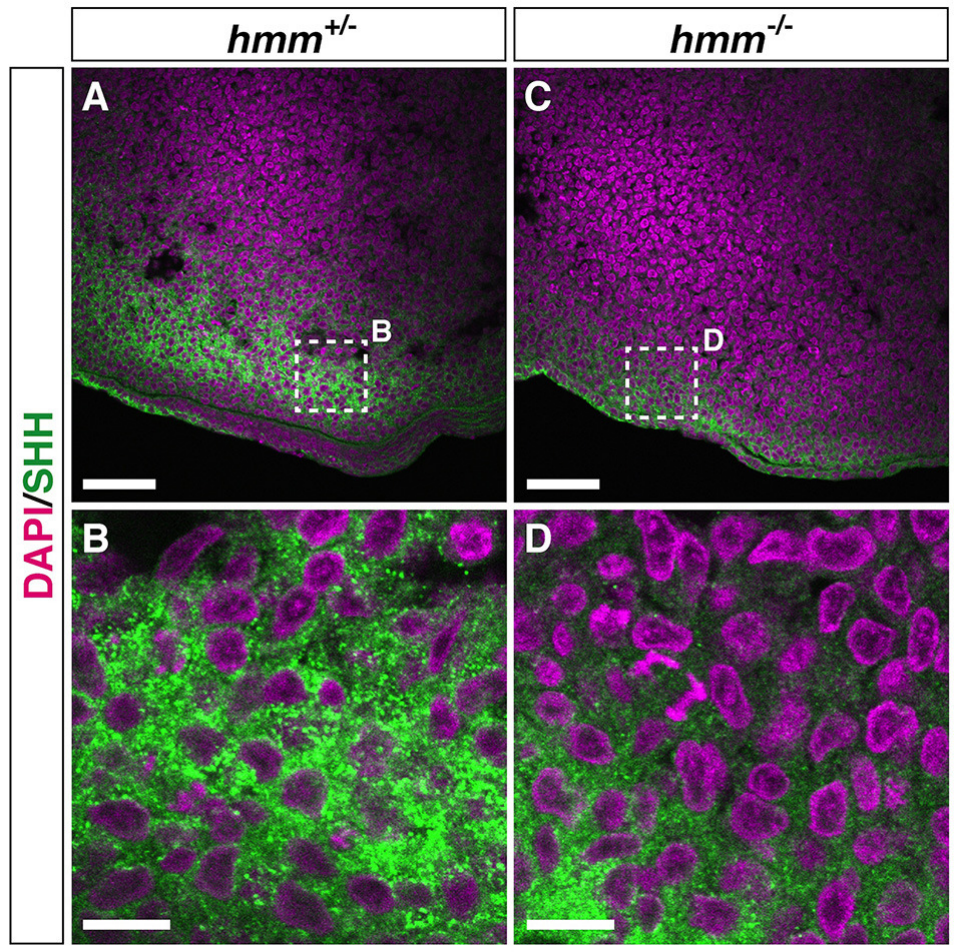
**SHH protein is detectable by immunohistochemistry in the ***hmm***^**−/−**^ limb bud**. Immunohistochemistry of SHH protein at the posterior limb bud of the St. 23 embryo is shown with fluorescent green. The nucleus is stained with DAPI. Frontal sections are oriented with the anterior side up and the posterior side down. **(B,D)** Higher magnification of the area enclosed by the dotted square in **A** and **C**. Scale bars, 50 μm **(A,C)**, 10 μm **(B,D)**.

We next examined the presence of SHH protein using immunohistochemistry. We found that the protein was detectable by anti-SHH antibody in the limb bud and the notochord in both *hmm*^+/−^ (Figures 6A,B, Figure S1B) and *hmm*^−/−^ (Figures 6C,D, Figure S1B) embryos. These observations raise the following possibilities in the *hmm*^−/−^ embryo: first, the secretion pathway of the SHH protein is defective; second, the SHH protein is dysfunctional; and third, the SHH signaling pathway in the target tissues is disrupted.

### High expression of GLI3A is observed in the presence of primary cilia in *hmm^−/−^* limb bud

**Figure 7:**
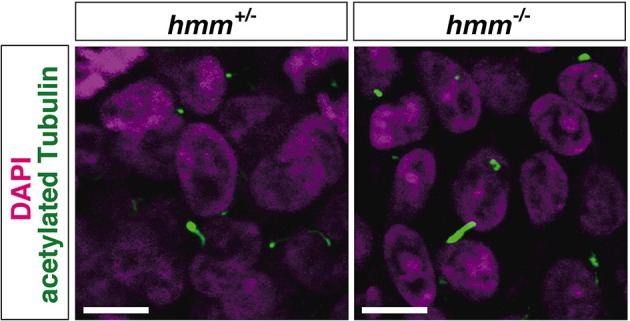
**The primary cilium is present in ***hmm***^**−/−**^ limb bud**. Immunohistochemistry of acetylated tubulin in the limb bud cells at St. 23 is shown with fluorescent green. The nucleus is stained with DAPI. Scale bars, 10 μm.

**Figure 8:**
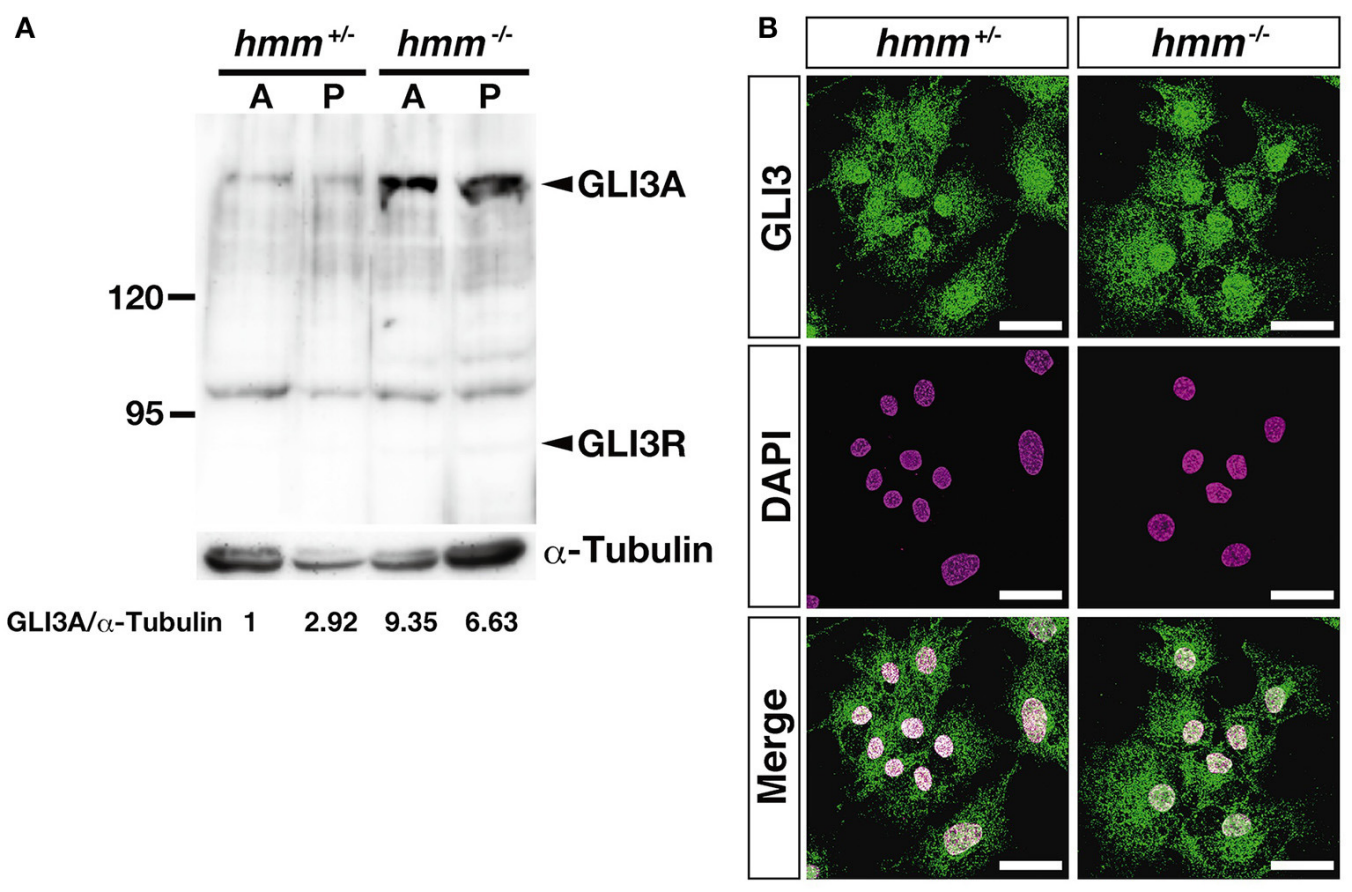
**High amounts of GLI3A protein are expressed in the ***hmm***^**−/−**^ limb bud. (A)** Western blotting of GLI3 protein and α-tubulin in the limb is shown. A and P indicate samples extracted from anterior and posterior limb buds. Relative ratio of GLI3A/α-Tubulin is shown at the bottom. **(B)** Immunohistochemistry of GLI3 protein in primary fibroblasts is shown with fluorescent green. The nucleus is stained with DAPI. Scale bars, 30 μm.

It was recently reported that loss of primary cilia induces constitutive activation of SHH signaling through high expression of GLI3A in both *talpid*^2^ and *talpid*^3^ mutants (Davey et al., 2006; Chang et al., 2014). Therefore, we visualized the primary cilia using immunohistochemistry targeting acetylated tubulin. However, we found that primary cilia were still present in both the *hmm*^+/−^ and *hmm*^−/−^ limb buds (Figure 7), suggesting that loss of primary cilia is not a cause of the *hmm*^−/−^ phenotype. We next performed western blotting for the GLI3 protein (Figure 8A), and found that the active form of GLI3, GLI3A, was highly expressed in both the anterior and posterior halves of the *hmm*^−/−^ limb bud compared to the *hmm*^+/−^ limb bud. In particular, high expression of GLI3A was observed on the anterior side of the *hmm*^−/−^ limb bud where *Shh* is not expressed. Expression of GLI3R was not detected in our experiments. This result raises the possibility that GLI3A protein does not function normally in the *hmm*^−/−^ limb bud.

Finally, we examined whether transport of the GLI3 protein from the cytoplasm to the nucleus is disrupted in the *hmm*^−/−^ embryo using immunohistochemistry. We observed that GLI3 protein is localized to the nucleus in both *hmm*^+/−^ and *hmm*^−/−^ primary fibroblast cells (Figure 8B). Taken together, our results imply that despite the presence of GLI3 protein in the nucleus, SHH signaling is abolished in the *hmm*^−/−^ embryo due to a loss of function of the GLI3 protein.

## Discussion

In this study, we examined the developmental properties of the *hmm*^−/−^ limb bud. We found that the abnormalities of the *hmm*^−/−^ limb bud develop through a different mechanism than those of the *talpid*^2^ and the *talpid*^3^ limb buds (Table 2). The *hmm*^−/−^ embryo showed disruption of SHH signaling in both the limb bud and the neural tube as in *talpid*^3^ (Davey et al., 2006), whereas in the *talpid*^2^ limb bud constitutive activation of SHH signaling is observed (Caruccio et al., 1999). While the *hmm*^−/−^ limb bud shows a similar phenotype to the *talpid*^3^ limb bud in terms of the SHH signaling pathway in the cells, several phenotypes are different between them. Expression of ectopic *Hoxd13* was observed uniformly from the posterior to anterior mesenchyme in the *talpid*^3^ limb bud (Francis-West et al., 1995), whereas its expression was restricted posteriorly in the *hmm*^−/−^ limb bud as it is in the wild-type (Figures 3S,S′,T,T′). This phenotype is unique to the *hmm*^−/−^ limb bud compared to the *talpid*^2^ (Rodriguez et al., 1996) and *talpid*^3^ limb buds, indicating that the HMM mutant is a novel type of *talpid* mutant. It has been reported that the expression level of GLI3R regulates the *Hoxd13* expression pattern along the AP axis (te Welscher et al., 2002b). The expression of *Hoxd13* in the limb is downregulated in the absence of *Shh* expression (Ros et al., 2003) because only GLI3R is present. When GLI3R is expressed at half the wild-type level, expression of *Hoxd13* can be faintly observed at the posterior side of the limb (te Welscher et al., 2002b). In the *Gli3*^−/−^ limb bud, expression of *Hoxd13* is expanded to the anterior side due to a lack of GLI3R protein, the same as in *talpid*^3^ mutants (Davey et al., 2006). Based on these observations, one possible reason for the different expression domains of *Hoxd13* between the *talpid*^3^ and the *hmm*^−/−^ limb bud is that a lower amount of GLI3R is expressed in the *talpid*^3^ limb bud than the *hmm*^−/−^ limb bud due to the lack of primary cilia in the *taplid*^3^ mutant. This could result in a high expression level of GLI3A (Bangs et al., 2011) and thus expanded expression of *Hoxd13* to the anterior side. In contrast, the expression level of GLI3R in the *hmm*^−/−^ limb bud would be slightly higher than the *talpid*^3^ limb bud because the primary cilium is still present. These results imply that the expression level of GLI3R might be dependent on the presence or absence of the primary cilia among *talpid* family mutants. In our experiments, we could not detect GLI3R expression by western blotting (Figure 8A), suggesting the possibility that the antibody we used does not cross-react with quail GLI3R. Further study is needed to examine the expression level of GLI3R among *talpid* family mutants using different antibodies in the future.

**Table 2:** **Typical phenotypes of three talpid mutants in the limb bud**.

	***talpid^2^***	***talpid^3^***	**HMM mutant**
*Ptch1* expression	Expanded to the anterior border (Caruccio et al., 1999)	Uniformly expressed at very low level throughout mesenchyme (Lewis et al., 1999)	Similar to *talpid^3^*
ZPA graft	–	High level *Ptch1* was induced (Lewis et al., 1999)	*Ptch1* was not Induced
Anterior graft	Induced digit duplication without *Shh* expression (Caruccio et al., 1999)	*Ptch1* was induced (Lewis et al., 1999)	–
*Bmp2* expression	Expanded throughout AP axis (Caruccio et al., 1999)	Uniformly expressed under the AER along the AP axis (Francis-West et al., 1995)	Downregulated
*Hoxd13* expression	Expanded throughout AP axis (Rodriguez et al., 1996)	Expressed in the anterior mesenchyme (Francis-West et al., 1995)	Expressed only at the posterior side as same as wild-type
Primary cilia	Disrupted (Chang et al., 2014)	Lack of primary cilia (Bangs et al., 2011)	Exist
Responsible gene	*C2CD3*	*KIAA0586*	not identified
*Gli3* expression	Expanded to the posterior side (Caruccio et al., 1999)	Expanded to the posterior side (Lewis et al., 1999)	Similar to *talpid^2^* and *talpid^3^*
GLI3 protein	Functional high GLI3A expression(Chang et al., 2014)	No functional high GLI3A expression (Davey et al., 2006)	No functional high GLI3A expression
*Gli1* expression	Expanded to the antetior side (Caruccio et al., 1999)	Expressed very weakly throughout the limb except distally under the AER (Lewis et al., 1999)	Similar to *talpid^3^*
SHH protein	–	Wide spreaded than normal (Davey et al., 2006)	Expressed as same as wild-type
Skeletal pattern	Short broad radius and narrower ulna (Dvorak and Fallon, 1992)	Radius and ulna were fused (Ede and Kelly, 1964)	Indistinguishable same size of short radius and ulna
	Claw was formed in the leg (Litingtung et al., 2002)	Claw was not observed due to severe syndactyly (Bangs et al., 2011)	Claw was formed in some leg digits

We showed that the *hmm*^−/−^ limb bud partially retained AP polarity at the limb bud stage in the absence of SHH signaling (Figure 3). Previous reports in the mouse embryo suggested that the antagonization of *Hand2* by *Gli3* specifies anterior-posterior polarity of the limb bud at early stages before *Shh* expression starts (te Welscher et al., 2002a). After *Hand2/Gli3* specifies the anterior-posterior polarity in the limb bud, SHH expression in the ZPA establishes the anterior-posterior axis. We infer that immature AP polarity could be maintained downstream of the *Hand2*/*Gli3* system in the *hmm*^−/−^ limb bud.

In the *hmm*^−/−^ limb bud, target genes downstream of SHH signaling (*Hoxd13*, and *Bmp2)* were expressed at much lower levels than in the *talpid*^2^ and *talpid*^3^ limb buds (Figures 3, 4, Table 2). The *hmm*^−/−^ limb bud showed severe lack of SHH signaling activity despite high expression levels of the activator form of GLI3, GLI3A. We therefore assume that the function of GLI3 as a transcriptional factor is affected in the *hmm*^−/−^ limb bud. Previous work has shown mice with a conditional knockout of *Sufu* in the limbs have polydactyly with severe hypoplasia of the humerus, distal phalanges, and a short radius and ulna. In addition, a high level of GLI3A expression was observed in the *Sufu*^−/−^ limb bud (Zhulyn and Hui, 2015), but *Gli1* expression was downregulated (Zhulyn et al., 2014). These phenotypes are reminiscent of the *hmm*^−/−^ limb bud. Given that high levels of GLI3A expression were observed in the *hmm*^−/−^ limb bud, the regulatory system of GLI3A as mediated by proteins such as SUFU might be disorganized in the *hmm*^−/−^ limb bud. However, we examined expression of *Sufu* by *in situ* hybridization, and the expression appeared to be the same in both the *hmm*^+/−^ and *hmm*^−/−^ limb buds (data not shown). The binding of SUFU to the GLI3A/KIF7 complex leads to GLI3R formation, but this is inhibited by the SMO/KIF3A/β-ARRESTIN complex in the presence of SHH, resulting in GLI3A formation (Kovacs et al., 2008). These results suggest that the function of SUFU protein might be disorganized in the *hmm*^−/−^ limb bud. When we examined the coding sequence of *Gli3* derived from reverse-transcribed mRNA from the *hmm*^−/−^ limb bud, we saw several abnormal splicing variants at the N-terminus and full-length ORF sequence of *Gli3* (data not shown). Prior work reports that the Polydactyly Nagoya (*Pdn*) mouse mutant has several abnormal splicing variants of *Gli3* at the N-terminus due to integration of a retrotransposon (Thien and Rüther, 1999). Homozygous *Pdn* mice show severe polydactyly. It is possible that the abnormal splicing variants of *Gli3* we observed in the *hmm*^−/−^ limb bud interfere with GLI3A activity as a dominant negative variant of GLI3.

On the other hand, our results indicate that SHH protein is dysfunctional in the *hmm*^−/−^ limb bud (Figure 5) even though it was detectable by immunohistochemistry (Figure 6). In contrast, SHH protein is functional in the *talpid*^3^ limb bud (Lewis et al., 1999) (Table 2). These results also suggest that the gene responsible for *hmm* is different from the cause of the *talpid*^3^ mutant. We suggest that post-translational modification of SHH protein might be disrupted in the *hmm*^−/−^ limb bud. After the full length of SHH protein is synthesized, autoproteolytic cleavage is induced concomitantly with cholesterol modification of the N-terminal region of SHH protein (SHH-N). After that, SHH-N is palmitoylated by skinny hedgehog (SKI) (Briscoe and Thérond, 2013) and secreted from the cells. When we examined the coding sequence of *Shh* expressed in the *hmm*^−/−^ limb bud, it was normal. Further, ZPA derived from the *hmm*^−/−^ limb bud did not induce expression of *Ptch2* near the implanted region (Figure 5D). These results imply that the secretion of SHH-N protein might be disrupted by a defect in palmitoylation. Future work needs to determine if SHH-N is palmitoylated in the *hmm*^−/−^ limb bud.

Altogether, our results suggest that both SHH secretion and GLI3 function are disrupted in the HMM mutant. Interestingly, mice lacking both *Shh* and *Gli3* show similar phenotypes in both the limb bud and neural tube. The *Gli3*^−/−^ limb bud still has normal expression levels of *Ptch1*, whereas the *Shh*^−/−^; *Gli3*^−/−^ limb bud shows no expression (Litingtung et al., 2002). The *hmm*^−/−^ limb bud showed very low expression of *Ptch1* (Figure 4). This phenotype is more similar to the *Shh*^−/−^; *Gli3*^−/−^ limb bud rather than the *Gli3*^−/−^ limb bud, suggesting that the *hmm*^−/−^ limb bud is not just caused by *Gli3* deficient conditions. Furthermore, expression of *Hoxd13* is not observed in the *Shh*^−/−^ limb bud, whereas the *Shh*^−/−^; *Gli3*^−/−^ limb bud has high expression of *Hoxd13*. The *hmm*^−/−^ limb bud also showed *Hoxd13* expression, suggesting that the *hmm*^−/−^ limb bud is not caused by just *Shh* deficient conditions. In terms of neural tube development, *Shh*^−/−^; *Gli3*^−/−^ mice show a milder dorsalization phenotype (Litingtung and Chiang, 2000; Persson et al., 2002) than *Shh*^−/−^ mice (Chiang et al., 1996; Pierani et al., 1999). This phenotype is like the one observed in the *hmm*^−/−^ neural tube (Figure S1). These observations support the idea that both SHH and GLI3 activity would be disrupted in the HMM mutant as in *Shh*^−/−^; *Gli3*^−/−^ mice. In the autopod, the function of GLI3 downstream of Indian hedgehog (IHH) is necessary for cartilage growth in the digits (St-Jacques et al., 1999). Therefore, the unidentifiable, malformed digits in the *hmm*^−/−^ limb bud likely result from the disruption of GLI3 function during both digit patterning and digital cartilage formation.

*Shh*^−/−^; *Gli3*^−/−^ mice show polydactyly with unidentifiable digits similar to the *hmm*^−/−^ limb bud. Recently, it was reported that *Gli3, Hoxa13*, and *Hoxd13* triple mutant mice show more severe polydactyly than *Gli3*^−/−^ mice (Sheth et al., 2012). Previous reports indicated that HoxA13 regulates cell adhesion in the chick limb bud (Yokouchi et al., 1991). It is possible that the cell adhesion molecule downstream of GLI3/HoxA13/HoxD13 is involved in the determination of digit number. In the *hmm*^−/−^ limb bud, expression of *Hoxd13* is expanded in the autopod (Figure 3) with a disruption of GLI3 function, resulting in irregularly spaced digital ray sequence (Figure 2). We propose that we can elucidate the mechanisms of regulating the distance between digital rays under the control of cell adhesion molecule using the HMM mutant. Furthermore, it was reported that the expression of the cell adhesion molecule N-CAM is altered in the *talpid*^2^ limb bud (Chuong et al., 1993), which implies that it contributes to the aberrant condensation and bunching of digits in this mutant (McGlinn et al., 2005). Thus, adhesion molecules are thought to be important for the pattern formation and cartilage differentiation of the digits in the autopod. Studying the HMM mutant could be especially useful for determining how cell adhesion plays a role in the development of digit number and morphology.

In conclusion, we revealed several molecular characteristics of the *hmm*^−/−^ limb bud that distinguish it from the *talpid*^2^ and *talpid*^3^ limb buds. These mutants all show the common phenotype of high amounts of GLI3A expressed in the limb, but the expression of SHH downstream target genes is unique in each mutant. We need further study to understand why SHH signaling is abolished in the *hmm*^−/−^ limb bud despite the presence of a high amount of GLI3A, and why the SHH protein is dysfunctional in the *hmm*^−/−^ limb bud. Several new SHH signaling components and their functions have been recently reported, including IFT proteins, the Cos2-Fu system, an enzyme for Hh processing, and regulators of GLI activity like DYRK2 and MAP3K10 (Ramsbottom and Pownall, 2016). These reports indicate that the SHH signaling pathway is more complex than previously thought. We propose that further analysis of the HMM mutant will provide new insight into the SHH signaling pathway. It can also serve as a useful model system for studying pattern formation like the *talpid*^2^ and *talpid*^3^ mutants did for vertebrate morphogenesis.

## Author contributions

YosM, MN, KK, AK, and TS conceived the project and designed the experiments. YosM performed gene-expression studies and the ZPA grafting; MN maintained HMM mutant; KK performed the immunohistochemistry. JF, KK, and TS. performed the OPT scanning. TS performed the western blot analysis. KA contributed the isolation of quail cDNA. YosM, MN, KK, MT, YoiM, AK, and TS wrote the paper.

## Funding

This work was supported by KAKENHI grant no. 25111710 and 25291050.

### Conflict of interest statement

The authors declare that the research was conducted in the absence of any commercial or financial relationships that could be construed as a potential conflict of interest.

## Supplementary material

### Supplemental information

*Shh* is also expressed at the notochord and floor plate, and determines the dorsal-ventral polarity of the neural tube. To check whether SHH signaling is disrupted in other tissues, we examined the expression pattern of genes downstream of SHH signaling in the neural tube (Figure S1A). In the *hmm*^−/−^ embryo, *Shh* was expressed in the notochord but not in the floor plate. *Ptch2* was expressed around the notochord in *hmm*^+/−^ embryo and in the ventricular zone located in the ventral neural tube. However, this expression was not observed in the *hmm*^−/−^ embryo, indicating that SHH signaling is disrupted in the neural tube as it is in the limb bud. We further checked the expression patterns of transcription factors that determine neuronal identity along the DV axis under SHH signaling (Davey et al., 2006). The expression patterns of dorsal markers *Pax3* and *Pax7* were not distinguishable between the *hmm*^+/−^ and *hmm*^−/−^ embryos. In contrast, expression of the intermediate marker *Pax6* was expanded ventrally in the *hmm*^−/−^ embryo. *Dbx2* is also expressed at an intermediate level in the *hmm*^+/−^ embryo, but its expression was reduced in the *hmm*^−/−^ embryo. *Islet1* expression was normally observed in the ventral spinal cord but not in the floor plate. It is notable that *Islet1* expression was expanded to the most ventral region of the spinal cord in the *hmm*^−/−^ embryo. These observations of dorsalization and downregulation of genes downstream of SHH indicate that SHH signaling is also disrupted in the spinal cord and neighboring tissue in *hmm*^−/−^ embryos.

Figure S1 **SHH signaling is reduced in the ***hmm***^**−/−**^ spinal cord. (A)** *In situ* hybridization of *Shh, Ptch2, Pax3, Pax7, Pax6, Dbx2*, and *Islet1* on the transverse sections of the spinal cord at St. 25. All images are oriented with the dorsal side up and the ventral side down. The red arrowhead in the *Pax6* figure indicates expanded *Pax6* expression at the ventral side. The yellow arrowhead in the *Dbx2* figure indicates the region where expression of *Dbx2* is downregulated. The red arrowhead in the *Islet1* figure indicates expanded *Islet1* expression at the floor plate. **(B)** Immunohistochemistry of SHH protein at the notochord and neural tube is shown with fluorescent green. The nucleus is stained with DAPI. Transverse sections are oriented with the dorsal side up and the ventral side down.
