# Supplementary figures and images for: Inactivation of Sonic Hedgehog Signaling and Polydactyly in Limbs of Hereditary Multiple Malformation, a Novel Type of Talpid Mutant
Source: Front Cell Dev Biol. 2016 Dec 27;4:149. doi: 10.3389/fcell.2016.00149 (PMC5187386; doi:10.3389/fcell.2016.00149)

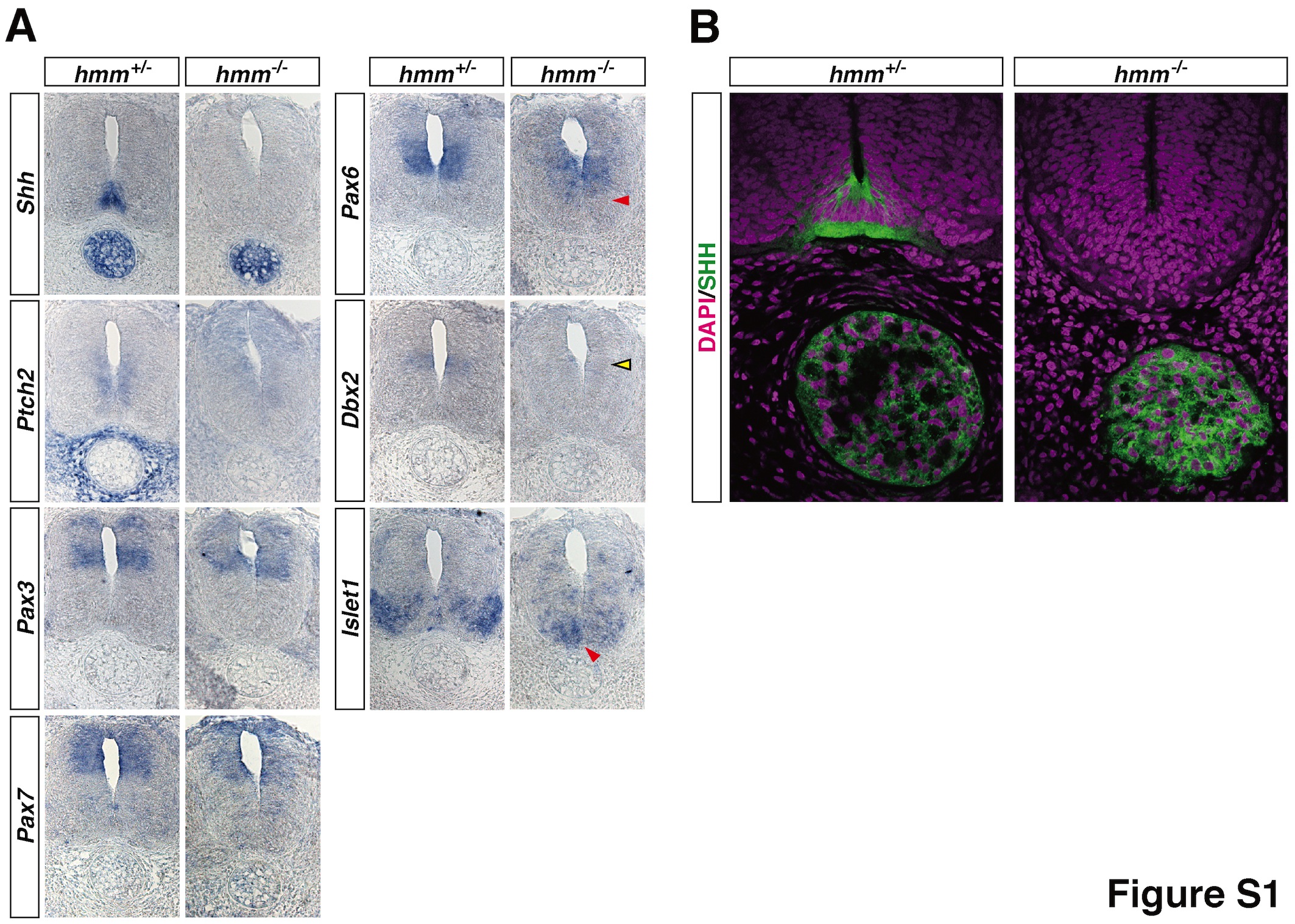

Supplement: Figure S1 — SHH signaling is reduced in the hmm−/− spinal cord. (A) In situ hybridization of Shh, Ptch2, Pax3, Pax7, Pax6, Dbx2, and Islet1 on the transverse sections of the spinal cord at St. 25. All images are oriented with the dorsal side up and the ventral side down. The red arrowhead in the Pax6 figure indicates expanded Pax6 expression at the ventral side. The yellow arrowhead in the Dbx2 figure indicates the region where expression of Dbx2 is downregulated. The red arrowhead in the Islet1 figure indicates expanded Islet1 expression at the floor plate. (B) Immunohistochemistry of SHH protein at the notochord and neural tube is shown with fluorescent green. The nucleus is stained with DAPI. Transverse sections are oriented with the dorsal side up and the ventral side down. [file Image1.JPEG]
